# Competition and coordination in Swedish botanical publication, 1820–79: Eleven editions of Hartman’s *Handbook*

**DOI:** 10.1177/0073275320987414

**Published:** 2021-02-11

**Authors:** Jenny Beckman

**Affiliations:** Uppsala University, Sweden

**Keywords:** History of scientific publication, history of botany, Sweden, nineteenth century, scientific authorship, history of education

## Abstract

In 1820, a *Handbook of the Flora of Scandinavia* by Carl Hartman was published in Stockholm by Zacharias Haeggström. The *Handbook* was a successful project for both author and publisher: similar enough to textbooks and academic publications to appeal in educational settings, yet ostensibly written for the general public. The *Handbook* went through eleven editions, becoming the standard reference flora for Swedish botanists – academic as well as others – before being succeeded after 1879 by a range of specialized floras aimed at schoolboys, students, or academic botanists. The trajectory of Hartman’s *Handbook* through the nineteenth century highlights the changing conditions of Swedish botanical publication. It draws attention to authorship as a scientific career tool, and, conversely, the significance of scientific texts in the emergence of commercial publishing in the first half of the nineteenth century.

## Introduction

At his death in 1849, Carl Johan Hartman was eulogized in the *Transactions of the Royal Swedish Academy of Sciences* for his achievements as “an author, a calling to which he devoted every available moment, with restless energy, until his last days.” His biographer praised his contributions to botany – which had earned him membership of the academy – while his activities as a physician were tolerantly described as “causing no disruption to his writing career,” despite medicine being his main occupation and source of income.^1^

Carl Johan Hartman’s enduring credit as an author was his *Handbook of the Flora of Scandinavia*, first published in 1820. The eleventh and final complete edition was published in 1879, three decades after his death, and edited by his son Carl Hartman.^2^ Hartman’s *Handbook* runs like “a green garland through most of the nineteenth century,” in the words of Gunnar Eriksson, the foremost historian of Swedish botany.^3^ Eriksson’s imagery emphasizes the unifying qualities of the *Handbook*, drawing botanists of various backgrounds, occupations, and generations to a common standard. At the same time, each new revised edition aimed to adapt to developments in publication practices, education, and career opportunities, and the trajectory of the *Handbook* charts the changing conditions of Swedish botanical publication in the nineteenth century.

In this paper, I situate Hartman’s *Handbook* in the landscape of scientific publication in nineteenth-century Sweden, examining it in the context of competition in the small botanical book market. I will show that its success depended on its relation to other botanical genres: similar enough to academic, Latin floras to merit serious consideration among botanists; accessible enough to appeal to a wider audience inside and outside Swedish schools; and benefiting, in its many editions, from the burgeoning botanical periodicals. In its inclusiveness, it profited from the problems of publishing science in Sweden, where scientific publication was normally a matter of private or public subsidies. For its publisher, it provided profit and respectability; for its authors, a means of building and maintaining positions, however different, in the Swedish botanical community. I examine its role in Swedish botany primarily as a matter of commercial competition – parallel to previous scholarship focusing on its role in contemporary debates about Naturphilosophie and Linnaean classification.^4^

I approach the publication history of Hartman’s *Handbook* through reviews, official documents, and correspondence, foremost that between Carl Johan Hartman and his editor Zacharias Haeggström, from the first edition of the *Handbook* in 1820 to the fifth edition and Hartman’s death in 1849.^5^ During this time, Hartman and Haeggström gradually carved a place for the handbook among Swedish botanical publications. I conclude by outlining the trajectory of the *Handbook* after 1849, as it went through six more editions under the editorship of Hartman’s son Carl.

## Hartman’s *Handbook* between catalogs and textbooks

The first edition of the *Handbook* was a substantial volume. A lengthy preface, including references and instructions on collecting and preparing plants, was followed by a sixty-page introduction where the author explained the parts of plants, their classification, habitats, and seasons. The bulk of the book – almost 500 pages – consisted of descriptions of the plants of the Scandinavian peninsula, arranged first by class and order, second by genus, mainly according to the Linnaean system (with some modifications), and listing the characters, habitats, and localities of each species. There were no illustrations, apart from two diagrams showing the shapes of leaves and flowers.^6^

In his *Flora Lapponica* (1737) and *Flora suecica* (1745), Linnaeus presented the species of a region or a country according to his system of classification and nomenclature, providing synonyms, habitats, and localities. “Floras” have a long history in botany, ranging from bare lists of species to herbals and multivolume catalogs, yet are often discussed as a genre in their own right. The format of Linnaeus’s floras was followed by many generations of botanists, particularly in his native Sweden.^7^ Bettina Dietz has shown how the multiple editions and translations of Linnaeus’s central works, such as *Systema naturae* and *Species plantarum*, as well as his floras, depended on the additions, corrections, and modifications of many naturalists. While this practice established Linnaeus as the center of natural history, it also allowed naturalists to shape his works to fit new audiences and contexts.^8^

For Swedish readers, Carl Fredrik Hoffberg, a doctor, botanist, and contemporary of Linnaeus, published a brief guide to the Linnaean system and nomenclature in 1763, expanding it in two later editions with descriptions of the uses of Swedish plants for medicinal and economic purposes. Samuel Liljeblad’s *Utkast til en svensk flora* from 1791 was, in its turn, presented as a companion to Hoffberg’s Linnaean outline.^9^ In later editions, Liljeblad’s *Utkast* outgrew its predecessors as Liljeblad added species and simplified the Linnaean system. He addressed it to “beginners and perhaps farmers” who had little time for Latin, but it also appealed to the students Liljeblad was teaching at Uppsala University as “botanices demonstrator” in the absence of the professor of botany, Linnaean disciple Carl Peter Thunberg.^10^ Liljeblad published the book at his own expense, and it sold well enough to go to two further editions in 1798 and 1816.^11^

Through Liljeblad’s *Utkast* and its predecessors, Hartman’s *Handbook* may be regarded a direct descendant of the comprehensive botanical works of Linnaeus. While Hoffberg’s and Liljeblad’s books had, at least initially, been cast as companions to the Linnaean canon, Hartman began by announcing his cautious departure from certain Linnaean maxims and diagnoses, for the purposes of “ease and certainty” in plant examination, and of acquainting his readers with more recent scientific views. He was, however, careful to distinguish it from more advanced botanical works that required a more knowledgeable reader, not least by the fact that it was written in Swedish. “Nothing,” he wrote, “would have been easier than to turn this book into a learned work of several volumes,” but instead he aimed for “comprehensibility, exactitude, and brevity,” characterizing his book as a mere “index” compared to more voluminous and famous works.^12^ He still introduced the reader, at some length, to botanical science and its philosophical debates, and he described the book as a useful and up-to-date guide to plant examination for beginners.^13^ In the second edition, he further specified that it was “not intended for continuous reading, but for occasional use.”^14^

Hartman labeled his work a “handbook,” although it was frequently referred to as a “flora.” Hoffberg and Liljeblad had chosen to call their books a “guide” (*anwisning*) and an “outline” (*utkast*), respectively, shying away from direct comparison with the Linnaean model. Hartman’s “handbook” suggested its use as a practical manual as well as a work of reference.^15^ It shares its multiple roles with many other handbooks, as recently pointed out by Angela Creager, Mathias Grote, Elaine Leong, and their collaborators. Drawing on book history as well as the history of science, they explore handbooks and manuals across a wide range of disciplines, languages, and contexts, finding pamphlets, multivolume opuses, recipes, and encyclopedic tomes. But even though handbooks resist easy characterization as a genre, they differ from other educational genres, such as textbooks, in their focus on practical knowledge as well as in their different paths on the book market.^16^

The development of science textbooks in the course of the nineteenth century provides an important complement to the trajectory of the *Handbook*. Antonio García-Belmar and José Ramón Bertomeu-Sanchez have pointed out the diversity among authors, audiences, and purposes of chemistry textbooks in the period.^17^ By the second half of the century, textbooks were emerging as a distinct genre, reflecting new relationships between authors, publishers, readers, and, not least, the state in the shape of new educational institutions and regulations.^18^ Handbooks, although less explicitly tied to specific educational contexts, underwent similar changes.

Placing Hartman’s *Handbook* in a wider context of textbooks, catalogs, and handbooks, I examine it as a publishing product, rather than as an episode in the history of a specifically botanical “flora” genre. As a handbook, it resembled and competed with other handbooks, textbooks, and scholarly works in the emerging Swedish scientific book market. It was less ambitious than the Latin scholarly works of academic botanists, yet intervened in their debates; it appealed to students, but was unconstrained by the demands of teachers and curricula. The inclusive character of the *Handbook* reflects the persistently permeable boundaries of field botany, and the expanding Swedish community of botanists, both inside and outside of academic institutions.^19^ For its author and its publisher, the handbook as a product was particularly fit to benefit from these multiple audiences.^20^

## Swedish botanical publication in the nineteenth century

Publishing science in Sweden was a risky business. Until the early nineteenth century, printing and publishing in Sweden were strictly regulated via permits and privileges. The new liberal press laws of 1810 established the freedom of the press, abolished printing privileges, relaxed the regulations on translations, and allowed anyone to operate a print shop and to trade in books.^21^ In the following years a number of new businesses tried to take advantage of the new opportunities.^22^ Among them were several academics attempting to improve their meager incomes and uncertain prospects. Vilhelm Fredrik Palmblad, who bought the academic printing shop in Uppsala in 1810, published Romantic periodicals before becoming professor, first of history, then of Greek, selling off the business at a profit in the 1830s. Botanist Carl Adolph Agardh tried his fortune at printing, less successfully, in Lund. He eventually attained the botany chair at Lund University and continued his involvement in scientific publication in other capacities. Hartman’s publisher Zacharias Haeggström abandoned an academic career in oriental languages to open a printing shop in Stockholm in 1813.^23^

However, few publications were likely to make a profit at all, except religious texts, almanacs, and scandals.^24^ Despite his radical views on science and politics, the most reliable best-seller in Agardh’s catalog was an overview of the Swedish liturgy.^25^ The commercial publisher N. M. Lindh, best known for his production of cheap translated novels, also relied on religious literature. As university printer, Palmblad could rely on academic and official commissions, but he published sermons, periodicals, and, increasingly, textbooks at his own expense.^26^ Haeggström, too, built his catalog and reputation by publishing translations of international bestsellers, particularly books about Napoleon, but soon began specializing in handbooks and textbooks.^27^ His first products in this field were translations of predominantly German textbooks – a lucrative strategy since the Swedish press laws recognized only the rights of the translator, not the original author.^28^

Textbooks were, in fact, among the few scholarly publications at all likely to make a profit in early nineteenth-century Sweden. Jonathan Topham has emphasized the importance of textbooks in British publishing in the early nineteenth century, providing a relatively dependable audience for publishers as well as authors.^29^ As Ann Shteir and Anne Secord have shown, the English market for botany in the first half of the nineteenth century allowed authors and publishers to produce a variety of textbooks targeted at different audiences.^30^ The Swedish market for scientific publications was, unsurprisingly, smaller. Throughout the eighteenth century, conflicts over printing privileges had often focused on textbooks, but the abolishment of privileges made many of the classic texts stipulated in school curricula fair game for printers.^31^ Secondary education was still limited to few students, fewer schools, and very few subjects, and competition was fierce. Science, in particular, had a weak presence in Swedish secondary schools in the first half of the nineteenth century. Although physics and natural history were included in the instructions to the school statutes of 1820, they had no fixed place in the curriculum, and there were seldom funds for hiring permanent teachers.^32^ In Lund, Uppsala, and Stockholm, school science was often taught by academic professors to bolster meager salaries. Consequently, both books and teachers routinely crossed the boundaries between schools and universities.

The unsettled status of science in secondary schools matched the ambiguity of the *Handbook*, with its multiple uses and audiences. As Hartman’s publisher Haeggström put it: “It will be a good book for our secondary schools, when they have managed to raise themselves from their present savage state – until then, in the universities.”^33^ Haeggström and Hartman almost certainly envisioned the *Handbook* as a successor to Liljeblad’s *Utkast til en svensk flora*, the most successful botanical textbook of the previous generation. The last edition of Liljeblad’s book was published after his death in 1816, and was the result of collaboration between several young botanists, including future professors Elias Fries and Carl Adolph Agardh.^34^ However, Fries, Agardh, and their fellow academic botanists in the generations after Linnaeus continued publishing mainly in Latin, with some Swedish publications aimed at agricultural audiences.^35^ Like most books, their works were typically published for subscription, at the expense of the author, the printer, or occasionally other patrons.^36^ There was general agreement among scholars and publishers that the conditions for Swedish scholarly publications remained poor.^37^ In 1811, the chemist Berzelius complained that the potential readers of his own works were too few for publishing in Swedish, and, some years later, the printer and bookseller Nils Wilhelm Lundequist stated that only works of science printed in Latin or one of the great modern languages had any commercial prospects, unless intended for schools.^38^

The dominant Swedish scientific publication in the early nineteenth century remained the periodical *Transactions of the Royal Swedish Academy of Sciences*. It dated from 1739 and was, like many similar periodicals of its time, published in the vernacular rather than Latin, to make it more useful in accordance with the cameralist doctrine of the period.^39^ It relied on government-sanctioned subsidies, and the academy occasionally contributed to the funding of struggling commercial projects in Swedish – notably the lavishly illustrated but troublesome *Svensk botanik* (1802–43), a multivolume catalog of Swedish plants – and served as a hub of Swedish scientific publications.^40^ In the course of the nineteenth century, the scope of the *Transactions* – along with the academy itself – narrowed to focus on academic science rather than the broad range of topics and scholars included in the previous century. Although Swedish remained the official language, the *Transactions* eventually allowed papers in other European languages, as academy fellows increasingly sought their readers among scientists outside the country, rather than in the small Swedish scientific community.^41^

Despite the precarious conditions for publishing botany in Swedish, a commercial botanical journal was launched in 1839. *Botaniska notiser* was the project of Elias Fries, then recently appointed professor of botany at Uppsala University, and Alexis Lindblom, nominally professor of philosophy at Lund University but a botanist by training and inclination, bringing experience of editing and writing for a local liberal newspaper. They modeled their journal after *Flora oder Botanische Zeitung*, published in Regensburg since 1802, and similarly aimed to “expand the study of Botany as well as its interest and esteem” in Scandinavia by publishing primarily floristic and plant geographical papers as well as reviews, correspondence, and notices.^42^ The journal soon attracted papers from a wide range of Scandinavian botanists. Hartman was a regular contributor, and used its publications for revised editions of the *Handbook*.

Although launched as an independent project, *Botaniska notiser* was published by academic botanists for the greater part of its run, and received intermittent government funding, keeping afloat despite repeated interruptions and changes in management.^43^ Neither entirely academic nor commercial, *Botaniska notiser* illustrates the conditions for botanical publication in Sweden: benefiting in terms of funding and credentials from academic connections, but surviving as an enterprise by attracting subscribers and contributors outside academic circles. It adds to the picture of the strategies employed by editors of scientific periodicals in Britain and France in the early nineteenth century, as they navigated between political periodicals and academic publications bolstered by the funds of scientific societies.^44^

## Hartman’s *Handbook*: Competition and the business of revision

As recounted by his biographers, Carl Johan Hartman (1790–1849) discovered botany when he “happened to come across” Liljeblad’s *Utkast* as a young boy. Raised in the provincial town of Gävle to take over the family glazier’s business, he nevertheless gained permission to continue his education at Uppsala University, studying botany under Liljeblad himself.^45^ Aiming for a medical degree, he supported his studies as a private tutor in a series of well-to-do families in Uppsala and Stockholm. Alongside his medical studies and tutoring, and with the encouragement of his employers, he continued the pursuit of botany, building a network of friends and correspondents. Most important among these was Olof Swartz, Permanent Secretary of the Royal Swedish Academy of Sciences and curator of its natural history collections. Through Swartz, Hartman gained access to the academy library and collections, and in 1813 a travel grant to study the flora and fauna of northern Sweden, resulting in his first scientific publication, an account of his journey published in the academy *Transactions*.^46^ Obliged to resume his medical studies, he graduated in 1822 and practiced medicine in various positions before eventually returning to his birthplace, Gävle, as district medical officer in 1833.

Somewhere in the circles of young botanists and scholars in Stockholm he met Zacharias Haeggström, and wrote what was to become the first of many editions of the *Handbook of the Flora of Scandinavia* while still a student. Haeggström soon commissioned Hartman for translations of two older works in natural history (continuing his practice of publishing translations of foreign bestsellers), as well as another original work, the medical handbook *Husläkaren* (1828, *The Family Doctor*), which caused considerable debate among reviewers and fellow physicians, but appeared in three editions over seven years.^47^ The textbooks and, most importantly, the handbooks provided Hartman with a source of income during the lean years of studying, private tutoring, and precarious medical positions.

A commercial success for both Hartman and Haeggström, the *Handbook* nonetheless caused controversy on its first appearance. The critical reviews of the first two editions in 1820 and 1832 revolved around its offenses against Linnaean classification, its use of the Swedish language, and its merits as a textbook. Significantly, the most vociferous critics were both publishing botanical works of their own at the same time. Göran Wahlenberg, lecturer and eventually professor of botany in Uppsala, wrote a series of scathing, anonymous reviews of the 1820 *Handbook* while in the process of finalizing his own Latin *Flora svecica*, conceived as heir to Linnaeus’s own.^48^ The Uppsala printer Palmblad, who was taking a commercial chance on the *Flora*, also provided the forum for Wahlenberg’s disapproval: his own periodical *Svensk Literatur-Tidning*.^49^

The 1832 edition of the *Handbook* was critically reviewed by Carl Adolph Agardh, professor of botany at Lund University. He was publishing the second volume of his own Swedish botanical textbook in the same year, and objected strongly to the *Handbook* as precisely a “textbook” exposing students to its eclectic approach to botanical theories. However, he praised it as a “handbook” summarizing the field for the scientist: “it is careful; it is complete; it is not copied, but rather assimilated through the views of the author; and it meets a real need in our educational literature.”^50^ In the small Swedish botanical book market, with its precarious footing in schools, the *Handbook* threatened both textbooks and scholarly publications, and both reviewers had reason to fear for the success of their own, self-published works.

With each edition, the position of the *Handbook* among Swedish botanists seemed more secure. In contrast to Agardh’s criticism of the second edition, Hartman’s friend Johan Emanuel Wikström, professor of botany at the Royal Academy of Sciences, stated laconically: “The work is widely known and overwhelmingly used. A review is therefore superfluous.”^51^ However, the Swedish scholarly book market did not allow for complacency, and both Hartman and Haeggström worried about competition. When Hartman was working on the third edition, and simultaneously writing a textbook in natural history, he learned that Pehr Fredrik Wahlberg, acting professor of natural history at the Karolinska Institute of Medicine in Stockholm, was planning a textbook of his own. Realizing that two textbooks appearing at the same time would be “pointless,” both Wahlberg and Hartman were anxious to avoid a “collision.”^52^ Hartman was reluctant to abandon his book and suggested collaboration, but Wahlberg declined. In the event, Hartman modified his plans and settled for a revised and expanded version of his own earlier translation of a German textbook. Wahlberg’s book on fodder plants appeared in 1835, seemingly without conflict.^53^

Haeggström steadily cajoled and nagged Hartman to publish more, and more quickly. Hartman’s early translations of German textbooks remained successful in updated and expanded versions, but a projected “Flora Oeconomica” and a geography textbook never materialized, despite Haeggström’s coaxing.^54^

The *Handbook* remained a dependable item in the Haeggström catalog, and sold “most admirably in Lund, Uppsala, and Stockholm” (although Haeggström wrote sarcastically of boastful booksellers who “asked for 100 copies, orders 80, and sells 11 in a year”). New, revised editions appeared in 1832, 1838, and 1843, each edition selling slightly faster than the previous one.^55^ Haeggström paid respectably: in 1845, he offered Hartman 500 *Riksdaler* for a new edition, and 250 for subsequent printings.^56^ This was much more generous than the rate for textbook translations, but substantially less than the 3,000 *Riksdaler* offered by Haeggström’s competitors P. A. Norstedt & Söner for a German textbook in 1849, indicating the still precarious position of science in schools and universities.^57^

Haeggström’s firm solidified its reputation as a publisher of textbooks and handbooks, but the success of the *Handbook* tempted competitors. In 1843, a young student, Johan Daniel Högberg, published a “Swedish flora” to devastating reviews and accusations of plagiarizing Hartman. Haeggström was nevertheless concerned:

It is scandalous that Öberg, or Sjöberg, or whatever his name is, should have copied you; and it is not inconceivable that his book is being used, for I seem to note smaller sales of the 4th edition than the one before, although I cannot say for certain before I complete my sales accounts at Christmas.^58^

Högberg’s book was unsuccessful, but the range of botany books available to Swedish readers increased during the 1840s. The introduction of compulsory elementary education in 1842 increased the potential market for textbooks, and, in the following decade, new school statutes made botany mandatory for all secondary school students, although school science was slow to develop.^59^ New textbooks and several local floras appeared, spurring Haeggström and Hartman to capitalize further on the success of the *Handbook*. They issued two abbreviated versions: a textbook, or “botanology,” based on the introduction to the *Handbook* in 1843, and a “pocket flora” in 1846, intended for excursions at a mere 191 pages, and including Swedish plant names at Haeggström’s behest.^60^

When the *Handbook* first appeared, both its author and its publisher benefited from the precarious scientific book market and the shaky status of botanical education. Ostensibly aimed at botanical beginners, it could be used in schools even though it did not conform to a specific curriculum. Written in Swedish, it appealed to a wider audience than the Latin works published by academic botanists. But its flexibility also cast it as competition for a number of different kinds of publication: for Wahlenberg’s scholarly Latin flora, for Agardh’s academic textbook and for Wahlberg’s economic one, and for the growing number of local floras appearing on the Swedish market.

To Haeggström, the *Handbook* as a product, and Hartman himself as an author, were components in a business that relied on constantly updating its catalog of handbooks and textbooks to maintain long-term profitability in the emerging, but still unreliable, Swedish market for scientific publications.

My good friend! It is time to stop dawdling, and to take up your pen in earnest. Too long has she been idle. Remember how many years have passed, since we agreed on the plans for your great Natural Science, your Flora Oeconomica, etc. If you are to have time for anything other than publishing new editions of your works, you must hurry, because before you know it, you will have to think about a new edition of your Flora.^61^

Hartman continually revised the *Handbook* according to the comments of his fellow botanists, as well as the promptings of his publisher. This quasi-serial character of handbooks and textbooks was an asset to Haeggström, with recurring demand at the beginning of each school year or collecting season, and the potential for ever new and revised editions. Haeggström also applied the serial model to his *Library of Popular Science* (*Bibliothek i populär naturkunnighet*), fourteen volumes of translations of works such as the Bridgwater treatises, and published by subscription over several years. The *Library* improved his status among Swedish scientists, while also emulating the practice of serial publication among publishers of popular novels. By the mid-1840s, Haeggström’s was the largest publishing company in the country.^62^

The *Handbook* also increasingly relied on serial scientific publications – *Botaniska notiser* in particular – for new information on observations, classifications, and publications, as well as for advertising new editions. In its many editions, it resembled a serial publication itself – kin to the multiple forms of periodicals in the nineteenth century: from miscellanies to specialized collections of original papers; from serial monographs to weekly bulletins; from commercial enterprises to government and institutional periodicals; from semi-public newsletters to promotional publications.^63^ Toward the twentieth century, as Mathias Grote has pointed out, encyclopedic handbooks were often published serially as separate volumes or fascicles over time.^64^ The seriality of the *Handbook* also recalled the constantly updated and adapted editions of the Linnaean canon in the eighteenth century, but in Hartman’s hands, Linnaeus’s universal project was recast as a national endeavor.^65^

Hartman achieved both income and status in the botanical community through the *Handbook*. On its pages, he cultivated the botanical networks he had begun building as a student, paying lavish tribute to the contributions of fellow botanists, and organizing his own last edition in 1849 according to the system of his influential friend Elias Fries. As the standing of the *Handbook* improved, Hartman still declared his business to be “editing” rather than “reforming” botanical systematics, treading the line between textbooks and academic botany.^66^ His duties as a district medical officer kept him in Gävle, at some distance from Swedish academic centers at Uppsala, Lund, and Stockholm, and he maintained his position mainly through correspondence and authorship. Nevertheless, he reached a respectable position in the Swedish botanical community, was elected fellow of the Academy of Sciences in 1838, and attended the meetings of the Scandinavian Association for the Advancement of Science in 1840 and 1844.^67^

The authors of the growing number of botanical textbooks and local floras in the second half of the century rarely achieved such honors. In other respects, their botanical career prospects were more promising. As teachers in the expanding secondary school system, they had an income as well as an audience, and Hartman’s botanist sons Carl and Robert both followed this path.^68^

## Conclusion: Hartman’s *Handbook* at the crossroads

Carl Johan Hartman died in 1849, barely finishing the fifth edition of the *Handbook*. His son Carl completed it and carried it through six more editions. It was more in demand than ever: a sixth edition was published in 1854, with three more in the following decade and a tenth in 1870. By then, the *Handbook* had achieved standard status among Swedish botanists. Authors of province floras arranged their books in the manner of Hartman, and collectors used it to organize their herbaria.^69^ As the eleventh edition was prepared for print, Carl Hartman was contacted by Leopold Neuman, secretary of the Lund Botanical Society. The Society served as a hub for botanical specimen exchange in Sweden, and it published the most influential of several plant lists, used by botanists to estimate the value of their specimens.^70^ Neuman was anxious to make sure that the Society’s plant list conformed to the organization of the *Handbook*, and asked for advance copies of the new editions in order to adjust the plant list accordingly.^71^ The standards of practice established by the Lund Society were made to correspond with the arrangement of the *Handbook*.

An increasing number of Swedish field botanists participated in the *Handbook*, contributing botanical observations as well as expertise on particular genera or species, thus gaining an audience without the effort and expense of publishing a monograph.^72^ *Botaniska notiser* remained a crucial source of information, but editorial ill-health and lack of both time and money made its appearance erratic. During its repeated interruptions, contributors to the *Handbook* communicated directly with its authors, among them the impressively named Uppsala student Thorgny Ossian Bolivar Napoleon Krok, who approached Hartman with the offer of canvassing his fellow students for botanical observations for the next edition.^73^ Along with an extensive list of cited authors (not least from *Botaniska notiser*), contributing botanists were recognized in the preface – in 1879 particularly for their authorship of specialist sections.^74^ Carl Hartman, on the other hand, styled himself the “editor” of his father’s work, the “corrections and additions” increasing with each edition, and culminating in the “entirely reworked” eleventh edition of 1879.

Under Carl Hartman, the *Handbook* retained its inclusive character, appealing to botanists inside and outside academic institutions, as well as in schools. The school statutes of the 1850s initially added to the demand for a handbook such as Hartman’s, and doubtlessly contributed to its continued success. But increasingly specific regulation of the science curriculum made its comprehensive scope less appealing. In 1869, a commission was appointed to evaluate science teaching and science textbooks in Swedish secondary schools. Krok – conscientious contributor to the *Handbook*, now teaching science at a Stockholm secondary school – was tasked with the section on botany and zoology, together with Hartman himself.^75^

Of the eight textbooks, fifteen floras, and four illustrated volumes evaluated, none gained full approval from Krok, the de facto author of the evaluation. Not even Hartman’s *Handbook*. Krok acknowledged its unparalleled position among Swedish floras and its great influence on later books: “Few floras have achieved such great trust, such wide use, or stimulated such large interest for descriptive botany as this.” However, his critical eye found many problems in the *Handbook*, in its account of characters, habitats, synonyms, sources; in what it included as well as what it left out.^76^

In their evaluation, the commissioners formulated a specific conception of the content and organization of school textbooks. But they also established “handbooks” as a separate category in all disciplines, subject to different requirements. Dictionaries differed from language primers, atlases from geography textbooks, collections of mathematical tables and scientific formulae from textbooks in physics and chemistry. In botany, a “flora” was the stipulated handbook.^77^ Hartman’s *Handbook* may have seemed a blessing to teachers in the 1850s, when the new school statutes suddenly required them to supervise the herbaria of their students, but by 1869 teachers and students were using local floras: less cumbersome and more affordable, specifically targeted at students and plant collectors of particular regions.^78^ (In fact, Carl Hartman’s local flora of the region surrounding Örebro, intended for his own students, was relatively favorably assessed by Krok.^79^)

Simultaneously too advanced and not comprehensive enough, Hartman’s *Handbook* now found itself sitting uneasily between categories. After the death of Carl Hartman in 1884, Krok himself attempted to take up the baton at the request of the family.^80^ His revised version was published in specialized installments and acclaimed by field botanists, but did not survive beyond the first few ambitious volumes; nor did a second attempt thirty years later.^81^ As envisioned by Hartman’s posthumous followers, the *Handbook* would resemble the encyclopedic late nineteenth-century chemistry handbooks examined by Mathias Grote. Often produced serially, and intended for practitioners rather than students, they differed not only from textbooks, but from the multipurpose volumes produced by the Hartmans.^82^

At the other end of the spectrum, Krok and fellow teacher Sigfrid Almquist (another key contributor to the final edition of the *Handbook*) published their own “Swedish flora” explicitly intended for schools.^83^ Although some critics found it too elementary, it was more in line with the requirements stipulated by Krok in his evaluation, and soon achieved an unparalleled position in Swedish secondary schools. The Krok–Almquist flora, too, was published by the Haeggström firm – but while textbooks remained central products, Zacharias’s son Ivar specialized in poetry and novels on the expanding Swedish book market of the late nineteenth century.^84^

The later editions of Hartman’s *Handbook* overlap with the early years of George Bentham’s *Handbook of the Flora of the British Isles*, which went through seven editions and revisions in the hands of several British botanists between 1858 and 1924. Bentham’s *Handbook* offers contrasts as well as parallels with Hartman’s. Like Hartman’s, it was originally addressed to “beginners” but eventually reached a wider readership, particularly after the addition of illustrations to the second edition. But it remained controversial among academic botanists, despite the credentials of Bentham and Sir Joseph Hooker, who revised it in 1887. David Allen ascribes its longevity, in the face of academic criticism, mainly to a lack of competition. British field botanists longed for a book that could combine the features of an examination manual, a flora for students, and a comprehensive catalog, and in the absence of alternatives, Bentham’s handbook filled the lacuna.^85^ In the same period, and in spite of its established position among academic botanists as well as students and collectors, the task of navigating between textbook and catalog stretched Hartman’s *Handbook* too far. As an author, the industrious Krok attempted to bridge the divide, but he did so through two separate publications.^86^

The trajectory of Hartman’s *Handbook* reflects changes in botanical education as well as in the Swedish book market. In its broad scope, it appealed to schoolboys, students, and established botanists, making it a profitable product in a precarious book market with a small scientific readership, particularly in Swedish, and a limited demand for textbooks. Its success continued as science education developed, in schools as well as universities, underpinning a vigorous and growing amateur community. Its demise marks the divergence of scientific genres and practices in the course of the nineteenth century, and partly – perhaps paradoxically – the strong position of botany in Swedish schools. By the second half of the nineteenth century, handbook authorship in botany was the path to school, rather than to the Royal Swedish Academy of Sciences, for books as well as authors. Despite Hartman’s substantial revisions to the eleventh edition following the criticism of the textbook commission, the *Handbook* lost its position in the profitable textbook market and was supplanted by local floras and specialized handbooks intended directly for schools and mostly written by secondary school teachers. The particular role assigned to “handbooks” in Swedish botanical education by the textbook commission was eventually filled by the Krok–Almquist flora for schools, to the great gain of its authors and publishers. Hartman’s *Handbook* had become the wrong kind of handbook.

## Footnotes

1. [Johan Emanuel Wikström], “Biografi öfver Carl Johan Hartman, medicinae doktor, provincial-läkare i Gestrikland, riddare af Kongl. Nordstjerne-Orden,” *Kungl. vetenskapsakademiens handlingar* (1849): 277–84. I have discussed Hartman’s *Handbook* specifically in the context of the history of education in a paper in Swedish, published as “Botanikens gränser: Om böcker, utbildning och karriärer,” in Peter Josephson and Thomas Karlsohn (eds.), *Universitetets gränser* (Göteborg: Arche, 2019), pp.38–60.

2. Carl Johan Hartman, *Handbok i Skandinaviens flora, innefattande Sveriges och Norriges vexter, till och med mossorna* (Stockholm: Zacharias Haeggström, 1820); Carl Johan Hartman and Carl Hartman, *C. J. Hartmans handbok i Skandinaviens flora: Innefattande Sveriges och Norges växter, till och med mossorna* (Stockholm: Haeggström, 1879). The editions in between appeared in 1832, 1838, 1843, and 1849 under Carl Johan Hartman; in 1854, 1858, 1861, 1864, and 1870–1 under his son Carl.

3. Gunnar Eriksson, “Johan Emanuel Wikström,” in Bengt Jonsell (ed.), *Bergianska botanister: Bergianska stiftelsen och dess professorer under första seklet* (Stockholm: Atlantis, 1991), pp.45–67, 50.

4. Gunnar Eriksson, *Elias Fries och den romantiska biologien* (Uppsala: Almqvist och Wiksell, 1962), pp.96–8; Hans Krook, “Göran Wahlenberg och Carl Johan Hartman: En episod från de svenskspråkiga florornas genombrottstid,” *Svensk botanisk tidskrift* 54 (1960): 220–8. Jonathan Topham describes his study of Cambridge mathematics textbooks from a publishing perspective as “parallel” to Andrew Warwick’s work on pedagogical practice. Jonathan Topham, “A Textbook Revolution,” in Marina Frasca-Spada and Nicholas Jardine (eds.), *Books and the Sciences in History* (Cambridge: Cambridge University Press, 2000), pp.317–37, 318.

5. The records of Haeggström’s business have been lost – an all-too-common fact of early Swedish publishing history, as told by Sven Rinman, *Studier i svensk bokhandel: Svenska bokförläggareföreningen 1843–1887* (Stockholm: Norstedt, 1951), pp.63–4, 472–3. A brief history of the company is Carl Forsstrand, *Hæggströmska boktryckeriet 1813–1863–1913: En återblick med anledning af hel- och halfsekeljubileet* (Stockholm: Ivar Haeggström, 1913).

6. A digitized version of the 1820 edition of Hartman’s *Handbook* is available at “Handbok i Skandinaviens flora, innefattande sveriges och norriges vexter, till och med mossorna,” <https://catalog.hathitrust.org/Record/008594662> (August 17, 2020). Hartman described the scope of the *Handbook* as the “plants of the Scandinavian peninsula” (p.3). All eleven editions of Hartman’s *Handbook* appeared during the period (1814–1905) when Sweden and Norway were joined in a political union. Although the *Handbook* was extensively used, particularly in Sweden and Swedish-speaking Finland, Norwegian culture remained resolutely separate and a comprehensive Norwegian handbook appeared in the second half of the nineteenth century. See Gunnar Eriksson, “Flora Writing in Norden,” in Bengt Jonsell (ed.), *Flora Nordica*. General volume (Stockholm: Bergius Foundation, Royal Swedish Academy of Sciences, 2004), pp.1–33, 19–22.

7. Alix Cooper discusses the delineation of local floras in *Inventing the Indigenous: Local Knowledge and Natural History in Early Modern Europe* (Cambridge: Cambridge University Press, 2007), while David G. Frodin gives a botanist’s perspective on the “evolution of floras” in *Guide to Standard Floras of the World: An Annotated, Geographically Arranged Systematic Bibliography of the Principal Floras, Enumerations, Checklists, and Chorological Atlases of Different Areas* (Cambridge: Cambridge University Press, 2001), pp.24–51. A historical overview of Nordic floras is Eriksson, “Flora Writing in Norden” (note 6), while Jan Thomas Johansson and Torbjörn Lindell focus on the biographies of Swedish flora writers: Jan Thomas Johansson and Torbjörn Lindell, “Från örtabok till Wahlenberg: Historien om svensk kärlväxtfloristik tar sin början,” *Svensk botanisk tidskrift* 88 (1994): 267–86; Jan Thomas Johansson and Torbjörn Lindell, “Från Agardh och Fries till 1800-talets mitt. Svensk kärlväxtfloristik tar fart,” *Svensk Botanisk Tidskift* 89 (1995): 345–63; Jan Thomas Johansson and Torbjörn Lindell, “Från det sena 1800-talet -Thedenius’ och Kindbergs tid. Svensk kärlväxtfloristik rullar på,” *Svensk Botanisk Tidskrift* 90 (1996): 33–48; Jan Thomas Johansson and Torbjörn Lindell, “Från Johansson till rutinventering. Svensk kärlväxtfloristik i modern tappning,” *Svensk Botanisk Tidskrift* 90 (1996): 177–97.

8. Bettina Dietz, “Contribution and Co-production: The Collaborative Culture of Linnaean Botany,” *Annals of Science* 69 (2012): 1–19; Bettina Dietz, “Linnaeus’ Restless System: Translation as Textual Engineering in Eighteenth-century Botany,” *Annals of Science* 73 (2016): 143–56. See also Staffan Müller-Wille, “Names and Numbers: ‘Data’ in Classical Natural History, 1758–1859,” *Osiris* 32 (2017): 109–28, on using the “data” of Linnaean catalogs for new scientific purposes.

9. Carl Fredrik Hoffberg, *Anwisning til naturens kännedom. Första delen. Om wäxt-riket. Efter herr arch. och riddaren von Linnès botaniska grunder* (Stockholm: Johan Georg Lange, 1768); Samuel Liljeblad, *Svenska ört-slagen, eller Kort afhandling, om sättet at efter botaniske grunder urskilja svenska växterna, til classer, ordningar och slägter* (Upsala: Joh. Edmans enka, 1791); Samuel Liljeblad, *Utkast til en svensk flora, eller Afhandling om svenska växternas väsendteliga kännetekn och nytta* (Upsala: Joh. Edmans enka, 1792). Both Hoffberg’s *Anwisning* and Liljeblad’s *Utkast* ran to three editions.

10. Quotations are from the preface to the second edition. Samuel Liljeblad, *Utkast til en svensk flora, eller Afhandling om svenska växternas väsendteliga kännetecken och nytta*. Andra uplagan, tilökd och förbättrad (Upsala: Joh. Fr. Edman, k:gl. ac. boktr. 1798), s. p. [iv]. Elias Fries would wistfully recall it as the textbook of his generation. Elias Fries, *Botaniska utflygter: en samling af strödda tillfällighetsskrifter*, Vol. III (Stockholm: Hæggström, 1864), p.82.

11. Olle Franzén, “Samuel Liljeblad,” *Svenskt biografiskt lexicon*, Vol. XXIII (Stockholm: Svenskt biografiskt lexikon, 1980–1), p.4; Gunnar Eriksson, *Botanikens historia i Sverige intill år 1800* (Uppsala: Almqvist & Wiksell, 1969), pp.265, 302.

12. Hartman, *Handbok* (1820), p.5 (note 2).

13. Hartman, *Handbok* (1820), p.5 (note 2).

14. Carl Johan Hartman, *Handbok i Skandinaviens flora, innefattande Sveriges och Norriges vexter, till och med mossorna* (Stockholm: Zacharias Haeggström, 1832), p.vi.

15. See also Ann B. Shteir, “Bentham for ‘Beginners and Amateurs’ and Ladies: Handbook of the British Flora,” *Archives of Natural History* 30 (2003): 237–49, 238, on the respective claims of a “flora,” a “handbook,” and a “manual” in later nineteenth-century British botany.

16. Angela Creager, Mathias Grote, and Elaine Leong, “Learning by the Book: Manuals and Handbooks in the History of Science,” *British Journal of the History of Science: Themes* 5 (2020):1–13. The definition given in the *Swedish Academy Dictionary* in 1930 is similarly inclusive, ranging from a “book (particularly of a small size) intended for frequent use” to “large textbook” and “encyclopedia.” The examples, from 1538 to 1910, suggest increasing size as well as range. *Svenska Akademiens ordbok* (Lund: Gleerup, 1893–), Vol. XI (1930), column H 249.

17. Antonio García-Belmar and José Ramón Bertomeu-Sanchez, “French Chemistry Textbooks, 1802–1852: New Books for New Readers and New Teaching Institutions,” in Anders Lundgren and Bernadette Bensaude-Vincent (eds.), *Communicating Chemistry: Textbooks and their Audiences, 1789–1939* (Canton, MA: Science History Publications, 2000), pp.19–56. A famous example of a textbook used as a vehicle for publishing new results is analyzed in Michael Gordin, “The Textbook Case of a Priority Dispute: D. I. Mendeleev, Lothar Meyer, and the Periodic System,” in Jessica Riskin and Mario Biagioli (eds.), *Nature Engaged: Science in Practice from the Renaissance to the Present* (New York: Palgrave Macmillan, 2012), pp.59–82.

18. Antonio García-Belmar and José Ramón Bertomeu-Sanchez, “Atoms in French Chemistry Textbooks during the First Half of the Nineteenth Century,” *Nuncius* 19 (2004): 77–119; also Kathryn M. Olesko, “Science Pedagogy as a Category of Historical Analysis: Past, Present, and Future,” *Science and Education* 15 (2006): 863–80.

19. Sweden, alongside Britain and the United States, has a history of substantial amateur participation in floristic botany. I discuss amateur botany in Sweden, particularly its relation to secondary education and supposed “Linnaean traditions,” in Jenny Beckman, “Collecting Standards: Teaching Botanical Skills in Sweden, 1850–1950,” *Science in Context* 24 (2011): 239–58. The literature on amateur science is large and growing; for overviews of amateur botany in the United Kingdom and the United States, see David Elliston Allen, *The Naturalist in Britain: A Social History* (London: Allen Lane, 1976); Elizabeth B. Keeney, *The Botanizers: Amateur Scientists in Nineteenth-Century America* (Chapel Hill: University of North Carolina Press, 1992).

20. Thus I focus on the producers, rather than the users, of the *Handbook* in the communication circuit; cf. Robert Darnton, “What is the History of Books?” *Daedalus* 111 (1982): 65–83. A practice-oriented perspective on the making of herbaria with the help, or hindrance, of textbooks and examination manuals, is Beckman, “Collecting Standards” (note 19).

21. An overview of the Swedish press laws in the period, although focusing mainly on the debate about the freedom of the press, is Thomas von Vegesack, *När ordet blev fritt: Den långa vägen till tryckfrihet* (Stockholm: Natur och kultur, 2003).

22. The most comprehensive overviews of the history of Swedish publishing are still Henrik Schück, *Den svenska förlagsbokhandelns historia*, 2 Vols. (Stockholm: Norstedt, 1923), and Rinman, *Studier i svensk bokhandel* (note 5). Swedish scholarship on the history of publishing has often focused on the relations between printers, publishers, and book traders, and how these categories have interacted and overlapped in changing legal and economic conditions.

23. On Palmblad, see Petra Söderlund, *Romantik och förnuft: V. F. Palmblads förlag 1810–1830* (Hedemora: Gidlunds, 2000); on Agardh’s printing business, see Ewert Wrangel, *Gamla studentminnen från Lund* (Stockholm: Isaac Marcus, 1918), pp.98–102, 140–1; on the academic careers of Haeggström and his business partner Anders Strinnholm, see Forsstrand, *Hæggströmska boktryckeriet* (note 5); Sven Rinman, *“Zacharias Haeggström,” Svenskt Biografiskt Lexikon*, Vol. XVII (Stockholm: Svenskt biografiskt lexikon, 1967–9), pp. 708–10; Birgitta Fritz, “Anders Magnus Strinnholm,” *Svenskt Biografiskt Lexikon*, Vol. XXXIII (Stockholm: Svenskt biografiskt lexikon, 2007–11), p.742.

24. Rinman, *Studier i svensk bokhandel*, p.15 (note 5); Söderlund, *Romantik och förnuft*, p.44 (note 23). For a contemporary account, see Nils Lundequist, *Stockholms stads historia, från stadens anläggning till närwarande tid i trenne delar* (Stockholm: Zacharias Haeggström, 1828–9), Vol. III, p.321.

25. Wrangel, *Gamla studentminnen från Lund*, p.100 (note 23).

26. Söderlund, *Romantik och förnuft*, pp.74–88 (note 23); on N. M. Lindh, see also Brigitte Mral, “Våld, moral och andeväsen: Romaner på Lindhs förlag 1800–1820,” in Lars Gustafsson (ed.), *Lindhs litterära industri: Ett tryckeri och förlag i Örebro* (Örebro: Örebro högskola, 1990), pp.65–82.

27. Rinman, “Zacharias Haeggström” (note 23); Forsstrand, *Hæggströmska boktryckeriet*, pp.15–17 (note 5).

28. Haeggström was not alone in seeing the advantages of translations, although publishers focused more on translated novels than textbooks. On the rights of translators and authors, see Schück, *Den svenska förlagsbokhandelns historia*, pp.349–56 (note 22); on translated novels as money-makers for new publishers in early nineteenth-century Sweden, see Mral, “Våld, moral och andeväsen” (note 26); Gunnel Furuland, *Romanen som vardagsvara: Förläggare, författare och skönlitterära häftesserier i Sverige 1833–1851 från Lars Johan Hierta till Albert Bonnier* (Stockholm: LaGun, 2007).

29. Topham, “A Textbook Revolution,” 318–20 (note 4).

30. Ann Shteir, *Cultivating Women, Cultivating Science: Flora’s Daughters and Botany in England, 1760–1860* (Baltimore: Johns Hopkins University Press, 1996); Anne Secord “Botany on a Plate,” *Isis* 93 (2002): 28–57.

31. On conflicts over printing privileges for schoolbooks in the eighteenth century, see Schück, *Den svenska förlagsbokhandelns historia*, pp.159–60 (note 22).

32. On science in Swedish schools in the nineteenth century, see Yngve Löwegren, *Naturaliesamlingar och naturhistorisk undervisning vid läroverken* (Stockholm: Föreningen för svensk undervisningshistoria, 1974); Sigurd Åstrand, *Reallinjens uppkomst och utveckling fram till 1878* (Uppsala: Föreningen för svensk undervisningshistoria, 1976).

33. Letter from Zacharias Haeggström to Carl Johan Hartman, November 29, 1832, Uppsala University Library.

34. On collaboration on the last edition of Liljeblad’s flora, see Eriksson, *Elias Fries och den romantiska biologien*, pp.100–1, 171 (note 4). The term “textbook” was used by Elias Fries, “Fortsättning. II. Linneanska tidehvarfvet,” in *Botaniska utflygter*, Vol. III (Stockholm: Zacharias Haeggström, 1864), pp.53–107, 82.

35. Notably botanist Anders Jahan Retzius’ “Flora oeconomica,” *Försök til en flora oeconomica Sveciæ eller Swenska wäxters nytta och skada i hushållningen* (Lund: Joh. Lundblad, 1806). Another example is Carl Adolph Agardh’s book on tobacco, *Några ord om tobaks odlingens förbättring* (Lund: Berlingska boktryckeriet, 1819).

36. Rinman, *Studier i svensk bokhandel*, pp.26–7 (note 5), citing a German traveler to Stockholm in the period 1817–20.

37. For a discussion of the emergence of English as a scientific language, at the expense of other languages, see Michael Gordin, *Scientific Babel: How Science was Done before and after Global English* (Chicago: University of Chicago Press, 2015). On the difficulties of publishing science in Swedish, see Jenny Beckman, “The Publication Strategies of Jöns Jacob Berzelius (1779–1848): Negotiating National and Linguistic Boundaries in Chemistry,” *Annals of Science* 73 (2016): 195–207; and Jenny Beckman, “‘Sections of One Great Institution’: Prizes, Publications, and Transnational Communication at the Royal Swedish Academy of Sciences in the Nineteenth Century,” *Nuncius* 34 (2019): 377–91.

38. On Berzelius’ problems with Swedish, see Beckman, “The Publication Strategies of Jöns Jacob Berzelius,” 202–3 (note 37); see also Lundequist, *Stockholms stads historia*, p.321 (note 24).

39. On the Royal Swedish Academy of Sciences and its publications, see Sten Lindroth, *Kungl. Vetenskapsakademiens historia 1739–1818*, 2 Vols. (Uppsala: Almqvist & Wiksell, 1967).

40. Funding from the academy, in its turn, depended on the sole printing privilege remaining after 1810: the almanac monopoly granted to the Royal Swedish Academy of Sciences in 1747. On the almanac monopoly and its significance for the funding of the academy, see Lindroth, *Kungl. Vetenskapsakademiens historia*, Vol. I, pp.823–67 (note 39); Johan Kärnfelt, Karl Grandin, and Solveig Jülich (eds.), *Knowledge in Motion: The Royal Swedish Academy of Sciences and the Making of Modern Society* (Gothenburg: Makadam, 2018). On academy funding for *Svensk botanik*, see Lindroth, *Kungl. Vetenskapsakademiens historia*, Vol. II, pp.434–41 (note 39); Gunilla Törnvall, *Botaniska bilder till allmänheten: Om utgivningen av Carl Lindmans Bilder ur Nordens flora* (Stockholm: Atlantis, 2013). Government support for scientific and pedagogical publications was eventually formalized as a regular entry in the budget of the Ministry of Education, “Anslag till resestipendier samt läroböckers och lärda verks utgifvande,” commonly called “lärdaverksanslaget,” and was used sporadically throughout the nineteenth century and more regularly until the 1960s. An overview of government publication subsidies is in *1945 års universitetsberedning 6. Den vetenskapliga publiceringsverksamheten, personal-, institutions- och stipendiefrågor m. m.; Det akademiska befordringsväsendet*. Statens offentliga utredningar 1951:9 (Stockholm: Svenska tryckeriaktiebolaget, 1951); on “lärdaverksanslaget,” see p.20.

41. On the language of the *Transactions* in the nineteenth century, see Beckman, “Sections of One Great Institution” (note 37).

42. Alexis Lindblom, “Anmälan,” *Botaniska notiser* 1 (1839): 1–2, 2. A brief outline of the history and content of *Botaniska notiser* is Gunnar Weimarck, “Botaniska Notiser 1839–1980 and Developments in Botany Reflected in its Contents,” *Botaniska Notiser* 133 (1980): 415–42. On Lindblom’s career, see Olle Franzén, “Alexis Lindblom,” *Svenskt biografiskt lexicon*, Vol. XXIII (Stockholm: Svenskt biografiskt lexikon, 1980–1), p.319. The Regensburg journal was published under the name *Botanische Zeitung* 1802–7, and *Flora oder Botanische Zeitung* from 1818. See Jan A. Leussink, “A Short History and Bibliographical Analysis of the Journal *Flora* (Regensburg) (Vol. 1–71, 1818–1888). Part 1. The Editors,” *Taxon* 30 (1981): 375–92.

43. Weimarck, “Botaniska Notiser,” 416–18 (note 42). Lindblom was a regular applicant to the government funding for scientific publications managed by the Royal Swedish Academy of Sciences. Minutes of the Royal Swedish Academy of Sciences, November 8, 1843; December 13, 1843; October 10, 1844, Archives of the Royal Swedish Academy of Sciences, Stockholm.

44. Jonathan Topham, “Science, Print, and Crossing Borders: Importing French Science Books into Britain, 1789–1815,” in David N. Livingstone and Charles W. J. Withers (eds.), *Geographies of Nineteenth-Century Science* (Chicago: University of Chicago Press, 2011), pp.311–44; Jonathan Topham, “Anthologizing the Book of Nature: The Circulation of Knowledge and the Origins of the Scientific Journal in Late Georgian Britain,” in Bernard Lightman, Gordon McOuat, and Larry Stewart (eds.), *The Circulation of Knowledge between Britain, India, and China: The Early-Modern World to the Twentieth Century* (London: Brill, 2013), pp.120–52; Alex Csiszar, *The Scientific Journal: Authorship and the Politics of Knowledge in the Nineteenth Century* (Chicago: University of Chicago Press, 2018); Iain P. Watts, “‘We Want No Authors’: William Nicholson and the Contested Role of the Scientific Journal in Britain, 1797–1813,” *The British Journal for the History of Science* 47 (2014): 397–419. On the situation for a society publication such as the *Philosophical Transactions of the Royal Society*, see Aileen Fyfe, “Journals, Learned Societies and Money: *Philosophical Transactions*, ca. 1750–1900,” *Notes and Records* 69 (2015): 277–99. In a recent issue on editorship of scientific journals, Fyfe and Anna Gielas suggest that a transnational perspective on scientific journals may challenge categories such as commercial or academy journals. Aileen Fyfe and Anna Gielas, “Introduction: Editorship and the Editing of Scientific Journals, 1750–1950,” *Centaurus* 62 (2020): 5–20.

45. The quotation comes from [Wikström], “Biografi öfver Carl Johan Hartman,” 278 (note 1). On Carl Johan Hartman’s biography, see Olle Franzén, “Carl Johan Hartman,” *Svenskt biografiskt lexikon*, Vol. XVIII (1969–71), pp.295–7.

46. Carl Hartman, “Beskrifning af Åreskutfjellet i Jämtland,” *Kungl. Vetenskapsakademiens Handlingar* (1814): 57–115; see also [Wikström], “Biografi öfver Carl Johan Hartman,” 279–80 (note 1).

47. Jean Claude Mien Mordant de Launay, *Handledning i den finare träd- gårds-skötseln för blifvande trädgårdsmästare och älskare af denna konst; af M. Mordant de Launay. Med ett kort tillägg af några finare trädgårdsfrugters odlingssätt. Ur almanach pour l’année 181; le bon jardinier, dédié et présenté à s.m. l’impératrice Joséphine.* Öfversättning af C.J. Hartman (Stockholm: Zacharias Haeggström, 1824); Johan Georg Hoffman, *Johan Georg Hoffmanns Populära naturkunnighet; det är: en korrt och för alla begriplig underwisning i de för hwar man nyttigaste och nödwändigaste kunskaper, som höra till naturens kännedom, till läsning för den olärda klaszen af medborgare, samt till lärobok för apologist- och folkscholor, utgifwen, efter den 22:dra, af Nicolai förbättrade, tyska upplagan, men, på utgifwarens begäran, ytterligare genomsedd, efter nyare åsigter lämpad och med en korrt sundhetslära tillökt, af C.J. Hartman* (Stockholm: Zacharias Haeggström, 1828); Carl Johan Hartman, *Husläkaren, eller Allmänna och enskilda föreskrifter i sundhetsläran, samt sjukdomslära, eller korrt anwisning att känna och rigtigt behandla de flesta i Swerige förekommande inre och yttre sjukdomar; till bruk för husfäder, pharmaceutici: och alla, som oexaminerade syszelsätta sig med medicinens utöfning* (Stockholm: Zacharias Haeggström, 1828). A scathing review was published in *Stockholms-Posten* 1829, pointing out the dangers of giving medical information to the public, but by Hartman’s death in 1849 the book was widely cited as a success and a boon to the people. *Stockholms-Posten* no. 57 (1829), no. 58 (1829), no. 83 (1829); *Stockholms dagblad*, August 30, 1849; *Norrköpings tidningar*, September 1, 1849.

48. The dispute, as it played out in the correspondence between Wahlenberg, Hartman, and botanists Elias Fries and Johan Emanuel Wikström, is covered in Krook, “Göran Wahlenberg och Carl Johan Hartman” (note 4); see also Eriksson, *Elias Fries och den romantiska biologien*, pp.96–8 (note 4). Wahlenberg’s book was subtitled “post Linnaeum edita,” signaling his adherence to Linnaean principles. Eriksson, “Flora Writing in Norden,” pp.7–8 (note 6).

49. Söderlund, *Romantik och förnuft*, pp.141, 144 (note 23).

50. C. A. Agardh, “Handbok i Skandinaviens flora” (review), *Svenska litteratur-föreningens tidning* 13 (1833): 193–206, 193; Carl Adolph Agardh, *Lärobok i botanik* (Malmö: Gleerup, 1829–32).

51. Johan Emanuel Wikström, *Årsberättelse om botaniska arbeten och upptäckter* (Stockholm: Kungl. Vetenskapsakademien, 1833), p.279.

52. The word is used by both writers. Letter from Pehr Fredrik Wahlberg to Carl Johan Hartman, September 4, 1835, Uppsala University Library; letter from Hartman to Wahlberg, September 12, 1835, Archives of the Royal Swedish Academy of Sciences, Stockholm. Letter from Hartman to Wahlberg, January 20, 1833, Archives of the Royal Swedish Academy of Sciences, Stockholm.

53. Carl Johan Hartman, *Utkast till populär naturkunnighet: Ett försök till lärobok för de lägre undervisningsverken* (Stockholm: Zacharias Haeggström, 1836). Hartman had been considering emulating Jacob Brand’s *Erster Unterricht in der Naturlehre*, a German textbook of 1820, itself a revised version of Joseph Uihlein’s *Kurzer Unterricht in der Naturwissenschaft für die Jugend* from 1810. Pehr Fredrik Wahlberg, *Anvisning till svenska foderväxternas kännedom* (Stockholm: Nordström, 1835). Wahlberg had been asked to write a textbook by the Minister of Education, but it is unclear whether this was in fact the same book.

54. Carl Hartman, *Utkast till populär naturkunnighet: Ett försök till lärobok för de lägre undervisningsverken* (Stockholm: Zacharias Haeggström, 1845).

55. Letter from Zacharias Haeggström to Carl Johan Hartman, December 24, 1834, Uppsala University Library.

56. Letter from Zacharias Haeggström to Carl Johan Hartman, no date [1845], Uppsala University Library.

57. Schück, *Den svenska förlagsbokhandelns historia*, pp.357–69 (note 22).

58. Letter from Zacharias Haeggström to Carl Johan Hartman, September 9, 1845, Uppsala University Library. Johan Daniel Högberg, *Svensk flora, innefattande Sveriges phanerogamvexter, med en kort, förberedande vextlära: För nybörjare utarbetad och utgifven* (Örebro: N. M. Lindhs boktryckeri, 1843); on the reception of Högberg’s flora, see Anders Bohlin, “Högbergs flora,” *Svensk botanisk tidskrift* 105 (2011): 189–90.

59. On science textbooks for elementary schools, see Magnus Hultén, “Scientists, Teachers and the ‘Scientific’ Textbook: Interprofessional Relations and the Modernisation of Elementary Science Textbooks in 19th Century Sweden,” *History of Education* 45 (2016): 143–68.

60. Carl Johan Hartman, *Utkast till botanologien, eller vextläran i allmänhet, med särskildt afseende på förf:s Handbok i Skandinaviens flora [. . .] med tvänne taflor* (Stockholm: Zacharias Haeggström, 1843); Carl Johan Hartman, *Svensk och norsk excursions-flora: Phanerogamer och ormbunkar* (Stockholm: Zacharias Haeggström, 1846). Two further editions of the excursion flora appeared in 1853 and 1860.

61. Letter from Zacharias Haeggström to Carl Johan Hartman, December 24, 1834, Uppsala University Library.

62. On the appeal of new editions of classical works for publishers as well as audiences, see Aileen Fyfe, “Publishing and the Classics: Paley’s *Natural Theology* and the Nineteenth-Century Scientific Canon,” *Studies in the History and Philosophy of Science* 33 (2002): 729–51. See also Nick Hopwood, Simon Schaffer, and Jim Secord, “Seriality and Scientific Objects in the Nineteenth Century,” *History of Science* 48 (2010): 251–85. On early translations and serializations of novels in Sweden, see Mral, “Våld, moral och andeväsen” (note 26), and Furuland, *Romanen som vardagsvara* (note 28), who also discusses the advantages of serial publication connected to different postal rates for journals compared to books. Haeggström donated the *Library* to the Academy of Sciences and reputedly received praise for this, but there is no record of an award in the Academy archives. Letter from Haeggström to Jöns Jacob Berzelius, December 19, 1846, Archives of the Royal Swedish Academy of Sciences, Stockholm. On the supposed recognition, see Forsstrand, *Hæggströmska boktryckeriet*, pp.26–7 (note 5). On the success of the Haeggström firm, see Schück, *Den svenska förlagsbokhandelns historia*, Vol. II, pp.372–3 (note 22).

63. The diversity is discussed in Topham, “Anthologizing the Book of Nature” (note 44); Csiszar, *The Scientific Journal* (note 44); Fyfe, “Journals, Learned Societies and Money” (note 44).

64. Mathias Grote, “Total Knowledge? Encyclopaedic Handbooks in the 20th Century,” *British Journal of the History of Science: Themes* 5 (2020): 187-203, 201.

65. As Bettina Dietz points out, Linnaeus’s works were “translated” into national projects in the eighteenth century as well. Dietz, “Linnaeus’ Restless System” (note 8); Dietz, “Competition and Co-production” (note 8).

66. Carl Johan Hartman, *Handbok i Skandinaviens flora, innefattande Sveriges och Norriges vexter, till och med mossorna; ordnade efter prof. Fries’ system* (Stockholm: Zacharias Hæggström, 1849). The quotations are from the preface written by Hartman’s son Carl, referencing his father’s correspondence. Carl Hartman, “Förord,” in Hartman, *Handbok i Skandinaviens flora* (1849), p.iv.

67. On the Association, see Nils Eriksson, *“I andans kraft, på sannings stråt-”: De skandinaviska naturforskarmötena 1839–1936* (Göteborg: Acta Universitatis Gothoburgensis, 1991).

68. Olle Franzén, “Carl Hartman,” *Svenskt biografiskt lexikon*, Vol. XVIII (Stockholm: Svenskt biografiskt lexikon, 1969–71), p.297. A notable example is Knut Fredrik Thedenius, author of several local floras and textbooks, starting as an apothecary and ending his career as science teacher at a prestigious Stockholm school; on Thedenius and botanical careers in the period, see Johansson and Lindell, “Från det sena 1800-talet” (note 7).

69. See Johansson and Lindell, “Från det sena 1800-talet” (note 7) for examples of province floras following the *Handbook*.

70. Beckman, “Collecting Standards,” 247 (note 19).

71. Letter from Leopold Neuman to Carl Hartman, February 20, 1879, Uppsala University Library.

72. Secondary school teachers, such as Carl Hartman himself and his Stockholm colleague Sigfrid Almquist, used the *Handbook* as a vehicle for original scientific publications. Letter from Sigfrid Almquist to Carl Hartman, January 21, 1878, Uppsala University Library.

73. Letter from Th. O. B. N. Krok to Carl Hartman, November 15, 1860, Uppsala University Library.

74. Hartman, *C. J. Hartmans handbok i Skandinaviens flora*, Preface (s. p.) (note 2).

75.
*Kommissionen för behandling af åtskilliga till undervisningen i matematik och naturvetenskap inom elementarläroverken hörande frågor, *Underdånigt betänkande. Afgifvet den 9 oktober 1871* (Stockholm: Norstedt, 1872).*

76. Kommissionen, *Underdånigt betänkande*, pp.167–70, 169. (note 75).

77. Kommissionen, *Underdånigt betänkande*, p.58. (note 75).

78. A list of Swedish province floras is in Åke Lundqvist, “Orientering i den svenska provinsfloristiken,” *Svensk Botanisk Tidskrift* 75 (1981): 303–12.

79.
*Kommissionen, *Underdånigt betänkande*, p. 171 (note 75).*

80. Olle Franzén, “Thorgny O B N Krok,” *Svenskt biografiskt lexikon*, Vol. XXI (Stockholm: Svenskt biografiskt lexikon, 1975–7), p.587.

81. Th. O. B. N. Krok, *C. J. och C. Hartmans Handbok i Skandinaviens flora: innefattande Sveriges, Norges, Finlands och Danmarks ormbunkar och fanerogamer*. 12. uppl. utgifven af Th. O. B. N. Krok (Stockholm: Kinberg, 1889). The Lund botanist Otto R. Holmberg initiated the second attempt, *Skandinaviens flora*, 4 parts (Stockholm: Norstedt, 1922–31).

82. Grote, “Total knowledge?” (note 64).

83. Th. O. B. N. Krok and Sigfrid Almquist, *Svensk flora för skolor*, 2 Vols (Stockholm: Zacharias Haeggström, 1883).

84. For a brief discussion of the reception and eventual success of the Krok–Almquist flora, see Beckman, “Collecting Standards,” 244 (note 19). On the later history of the Haeggström firm, see Forsstrand, *Hæggströmska boktryckeriet* (note 5), pp.29–38.

85. David E. Allen, “George Bentham’s Handbook of the British Flora: From Controversy to Cult,” *Archives of Natural History* 30 (2003): 224–36; see also Shteir, “Bentham for ‘Beginners and Amateurs’ and Ladies” (note 15).

86. David Allen notes that British attempts to replace Bentham’s book in the same period failed on account of their grandiose scale. Allen, “George Bentham’s Handbook,” 233 (note 85).

